# Correction to: Inhibitors of alanine racemase enzyme: a review

**DOI:** 10.1080/14756366.2018.1456716

**Published:** 2018-04-10

Azam M. and Jayaram U. Inhibitors of alanine racemase enzyme: a review. J Enzyme Inhib Med Chem. 2016;31:517–526.

When the above article was first published online, the below errors were introduced:

1. In Figure 6 caption and in the text, the word ‘alafosfalin’ was incorrectly written as ‘alaphosphin’.
2. On page 524, the word ‘thiadiazolidinone’ was incorrectly written as ‘thiadiazolinedione’.
3. The figures were incorrect which has now been corrected in the online version.

The authors apologise for this error.

